# Trans-Ethnical Shift of the Risk Genotype in the CETP I405V with Longevity: A Chinese Case-Control Study and Meta-Analysis

**DOI:** 10.1371/journal.pone.0072537

**Published:** 2013-08-15

**Authors:** Liang Sun, Cai-you Hu, Xiao-hong Shi, Chen-guang Zheng, Ze-zhi Huang, Ze-ping Lv, Jin Huang, Gang Wan, Ke-yan Qi, Si-ying Liang, Lin Zhou, Ze Yang

**Affiliations:** 1 The Key Laboratory of Geriatrics, Beijing Hospital and Beijing Institute of Geriatrics, Ministry of Health, Beijing, China; 2 Department of Neurology, JiangBin Hospital, Nanning, Guangxi, China; 3 Department of Cardiothoracic Surgery, Guangxi Maternal and Child Health Hospital, Nanning, Guangxi, China; 4 Yongfu Committee of the Chinese People’s Political Consultative Conference, Yongfu, Guangxi, China; 5 Beijing Youth Science and Technology Club, Beijing, China; Johns Hopkins University, United States of America

## Abstract

**Background:**

The I405V polymorphism of the cholesteryl ester transfer protein gene (*CETP*) has been suggested to be a protective factor conferring longevity in Ashkenazi Jews, although findings in other races are not supportive. This paper describes a case-control study and a meta-analysis conducted to derive a more precise estimation of the association between *CETP* 405V and longevity.

**Methods:**

We enrolled 1,021 ethnic Han Chinese participants (506 in the longevity group and 515 controls), then performed a meta-analysis that integrated the current study and previously published ones. Pooled odds ratios (OR) were calculated for allele contrasts, dominant and recessive inheritance models to assess the association between *CETP* 405V and longevity according to the ethnic stratification.

**Results:**

Our case-control data indicated that *CETP* 405V is a longevity risk allele in all genetic models (*P*_*additive*_=0.008; *P*_*dominant*_=0.008, OR_dominant_=0.673; *P*_*recessive*_=0.017, OR_recessive_=0.654) after adjustment for the apolipoprotein E (*APOE*) ε4 allele, body mass index and high-density lipoprotein cholesterol. A synergy was detected between 405V and *APOE* ε4 (*P*=0.001, OR=0.530). Eight studies were eligible for meta-analysis, which confirmed 405V is the risky allele against longevity in all genetic models: allele contrasts (OR=0.81, 95%CI=0.74-0.88), dominant model (OR=0.72, 95%CI=0.64-0.82) and recessive model (OR=0.80, 95%CI=0.67-0.96). After ethnic stratification, 405V remained a risk allele in East Asians but no significant association was found in Europeans or white Americans.

**Conclusion:**

Our case-control study suggests *CETP* 405V as a risk allele against longevity in Chinese. The meta-analysis suggests the involvement of *CETP* 405V is protective in Ashkenazi Jews but is a risk allele against longevity in the East Asian (Chinese) population.

## Introduction

The recent increase in world-wide aging has resulted in an unprecedentedly focused on human longevity by health researchers, policymakers and the general public [1]. The prevalence of centenarians in the industrialized world is currently 1 per 10,000 people and estimated to approach 1 per 5,000 in the near future [2]. Nearly 20% of the world’s oldest people live in China; and this is expected to increase to over 25% by 2050 [1]. China thus has the largest potential to benefit from investigations into longevity. Previous twin studies have suggested that around a quarter of factors conferring longevity are genetic [3], with the extremely old believed to possess beneficial genetic protective factors against ageing [4].

There are also thought to be difference in genetic contributions among ethnicities for common disorders and longevity, and particular racial or ethnic groups often demonstrate high levels of certain diseases or characteristics, such as type 2 diabetes in the Pima Indians. Accordingly, if a genetic association is discovered in one racial or ethnic background, it is necessary to confirm it in another to achieve worldwide benefit, although the confounding from population genetic structure definitely reduce the replication efficiency in case-control studies [5]. According to the specific racial/ethnic genetic background and lifestyle, it is necessary to confirm one previous discovery in different populations, even for the translation and benefit around the world.

In surviving age-related diseases such as cardiovascular disease, Alzheimer’s disease, diabetes mellitus, and cancer, dysfunctions of lipid metabolism have been shown to be epidemiologically relevant in centenarians [6–8]. Cholesterol metabolism in older subjects was reported to differ from that in younger subjects [9], and accordingly, much research has focused on lipid metabolism, inflammation [10], insulin/IGF-1 pathway [11], and oxidative stress [12]. The cholesterol ester transfer protein (CETP), a hydrophobic glycoprotein, promotes the transfer of excess cholesterol esters (CE) from vessel wall to the liver through the so-called reverse cholesterol transport (RCT) pathway [13,14].

*CETP* I405V, a functional single nucleotide polymorphism (SNP) substitutes valine (V) for isoleucine (I; “ancestral allele”; NCBI SNP rs5882; I405V), and was originally thought to be associated with the high-density lipoprotein-cholesterol (HDL-c) concentration and coronary heart disease status [15]. In 2003, association between I405V and longevity was observed in Ashkenazi-Jews with the VV genotype and V allele being more prevalent in centenarians and centenarian offspring [16]. 405V is also associated with lower CETP protein serum levels and activity with increasing HDL levels and particle sizes [17,18]. The results of subsequent replication studies in Europeans, white American sand East Asian populations are inconsistent, however, with the suggestion that 405V might be a risk allele against longevity in some races (reviewed in 19–24).

Because of the complexities of longevity, it is likely to be necessary to analyze large numbers of subjects to elucidate the associations with even a single polymorphism. To this end, we performed an association analysis between *CETP* I405V and longevity in a Chinese population, as the largest such research conducted in East Asia to date, followed by a meta-analysis of the literature on similar subjects.

## Materials and Methods

### Case-control study

#### Ethical approval of the research protocol

The Ethics Committee of Beijing Hospital, Ministry of Health approved the study protocol. All participants were informed and provided informed consent in writing. All clinical investigation has been conducted according to the principles in the Declarations of Helsinki.

#### Subject population

The current study was based on the Longevity and Health of Aging Population in Guangxi China study, which recruited 1021 eligible participants (479 men and 542 women) aged 20-105 years from urban and rural areas of Yongfu County, South China in 2008 and 2010. Yongfu County has been qualified as the longevity town by Geriatric Society of China in 2007. Trained and qualified personnel conducted the households and home visits. In consideration of the ethnic confounding, only the ethnic Han was included.

The longevity groups comprised 506 unrelated volunteers (mean age 92.45±3.57 years, 134 men and 372 women), including 16 centenarians (aged 100-109), 430 nonagenarians (aged 90-99) and 60 Octogenarians (aged 80-89). The geography and nationality matched younger control group consisted of 515 unrelated volunteers (aged 20-69, mean age 41.40±10.54 years, 345 men and 170 women) without longevity history (no lineal family members aged above 80 for 3 generations). Information on demographic variables, health status, health behavior and physical activity were obtained using a standardized questionnaire. The fasting serum and plasma samples were separated for the laboratory testing of lipid and glucose parameters according to the previously described methods [25].

#### Genotyping

The genomic DNA was extracted from peripheral blood leukocytes using a standardized salting-out procedure. The *CETP* I405V (rs5882) was detected by polymerase chain reaction based restriction fragment length polymorphism (PCR-RFLP) with *Rsa*I (New England Biolabs, USA). Briefly, genomic DNA was amplified by PCR in PTC-225 (MJ RESEARCH, USA) using designed primers as below: 5’-GCAGAACAGTACTGGCCAAGCAGCG-3’ and 5’-GCGGTGATCATTGACTGCAGGAAGCTCTGTA-3’, generating a 308-bp fragment. Optimum PCR amplification was achieved with 1×PCR buffer, 2 mM MgCl2, 0.15µM of each primer, 0.2 mM dNTP, 1.0 Unit Taq polymerase and 20 ng genomic DNA. The cycling program included: a denaturation step at 95°C for 5 min, 35 cycles of denaturation at 95°C for 50s, annealing at 62°C for 50s, extension at 72°C for 50s and then a final extension step at 72°C for 7 min. After digestion by *Rsa*I the wild-type I allele shows a 308-bp fragment while the mutation V allele shows 278 and 30 bp. The polymorphism of *APOE* was genotyped according to the method described elsewhere [26]. The accuracy of genotyping has been further confirmed by Sanger’s sequencing in 97 randomly selected cases.

#### Statistical analyses for the case-control study

Statistical Package for Social Sciences (SPSS; SAS Institute, Cary, NC, USA) Windows, version 12.0 was used. Allele frequencies were determined by gene counting and the fit for Hardy-Weinberg equilibrium (HWE) was verified using Chi-square goodness-fit test. Continuous variables were compared with One-way ANOVA or student *t*-test between groups. Chi-square test was used to compare the quantitative variables. Multiple logistic regression analysis was used to test the gene-gene interaction analysis. And the odds ratio (OR) was used to estimate the strength of association between variables, with the OR 95% confidence intervals. Two-sided P<0.05 was considered statistically significant. Power calculations were performed with the program Genetic Power Calculator (http://pngu.mgh.harvard.edu/~purcell/gpc/cc2.html) [27].

### Meta-analysis

#### Identification and eligibility of relevant studies

This study was performed according to the proposal of Meta-analysis of PRISMA statement [28,29]. Considering of the alias of I405V, we performed primary literature search in PubMed, MEDLINE and EMBASE systemically to identify all available articles regarding associations between longevity and *CETP*. Besides, to get the comprehensive information for the further race-stratified analysis, we simultaneously searched in the Chinese National Knowledge Infrastructure (CNKI) as far as possible to get the comprehensive information for the further race-stratified analysis, with the combination of the following medical subject headings (MeSH): (“CETP” OR “Cholesteryl ester transfer protein”) AND (“Longevity” OR “centenarians” OR “long-lived individuals”) AND (“I405V” OR “polymorphism” OR “variations”) from January 1, 1995 to March 1, 2013. The corresponding citations of the identified articles were also examined manually. Two independent investigators (L.S. and X.H.S.) reviewed the titles and abstracts of all selected articles for the evaluation of their eligible for inclusion in meta-analysis. The full-text of the article should be retrieved when abstract could not provide the enough information to judge whether inclusion. Studies have to meet all the following criteria: (1) The publication should be an observation study (case-control or cross-sectional designs) between *CETP* I405V and longevity, (2) with sufficient genotype data or other information that could help us to estimate the OR with 95%CI, (3) the genotype distribution in the total subjects satisfied the Hardy-Weinberg equilibrium (HWE), (4) when study subjects or data duplicated or had been published more than once, the most complete study was chosen, (5) the search was limited to English or Chinese language papers as recommended [30].

#### Data extraction and quality control assessment

Two investigators (L.S. and X.H.S.) independently extracted the data from eligible selected articles using a structured form and entered them into a database according to the pre-specified criteria. When there was disagreement during the extraction process, it was resolved by discussion or by a third investigator (C.Y.H.). The following data elements of each study was extracted: first author name, year of publication, numbers of genotyped cases and controls, racial/ethnic composition of the study population, age of cases and controls and genotype frequencies among cases and controls, stratified by race/ethnicity whenever possible. If the detailed genotype data could not be found in the article, we contacted the corresponding author by E-mail to obtain this information.

#### Statistical Analysis

Meta-analysis was performed for *CETP* I405V, stratifying by race/ethnicity. Hardy-Weinberg equilibrium in all the subjects for each study was examined using goodness-of fit test (Chi-square test). It was considered statistically significant when P<0.05. Studies deviated from HWE were removed. We examined allele contrasts model (V vs. I), dominant model ((VV+VI) vs. II) and recessive model (VV vs. (VI+II)). Because all the selected articles were case-control studies, we used the pooled odds ratios (ORs) as the metric of choice. Heterogeneity between-study was assessed by chi-based Q statistic and confirmed significant if P<0.10. The pooled OR was calculated by the fixed-effects model (Mantel-Haenzel) with no heterogeneity among studies, otherwise the random-effects model (evaluated by DerSimonian and Laird, or Peto [31–33]. I-squared was also calculated to represent the percentage of total variation across studies that is a result of heterogeneity rather than chance, with values less than 25% considered “low”, about 50% considered “moderate” and more than 75% considered “high”. Larger value indicates increasing heterogeneity. To evaluate the ethnic-specific effects, subgroup analysis was performed by ethnicity. To access the stability of the meta-analysis, one-way sensitivity analyses were carried out. The Begg’s funnel plots were performed to evaluated publication bias qualitatively; Begg’s test and Egger’s test were performed to assess publication bias quantitatively. All statistical analysis was conducted using STATA 10.0 software (StataCorp, College Station, TX, USA). Two-sided P<0.05 was considered statistically significant.

## Results

### Case-control study

**Figure 1:**
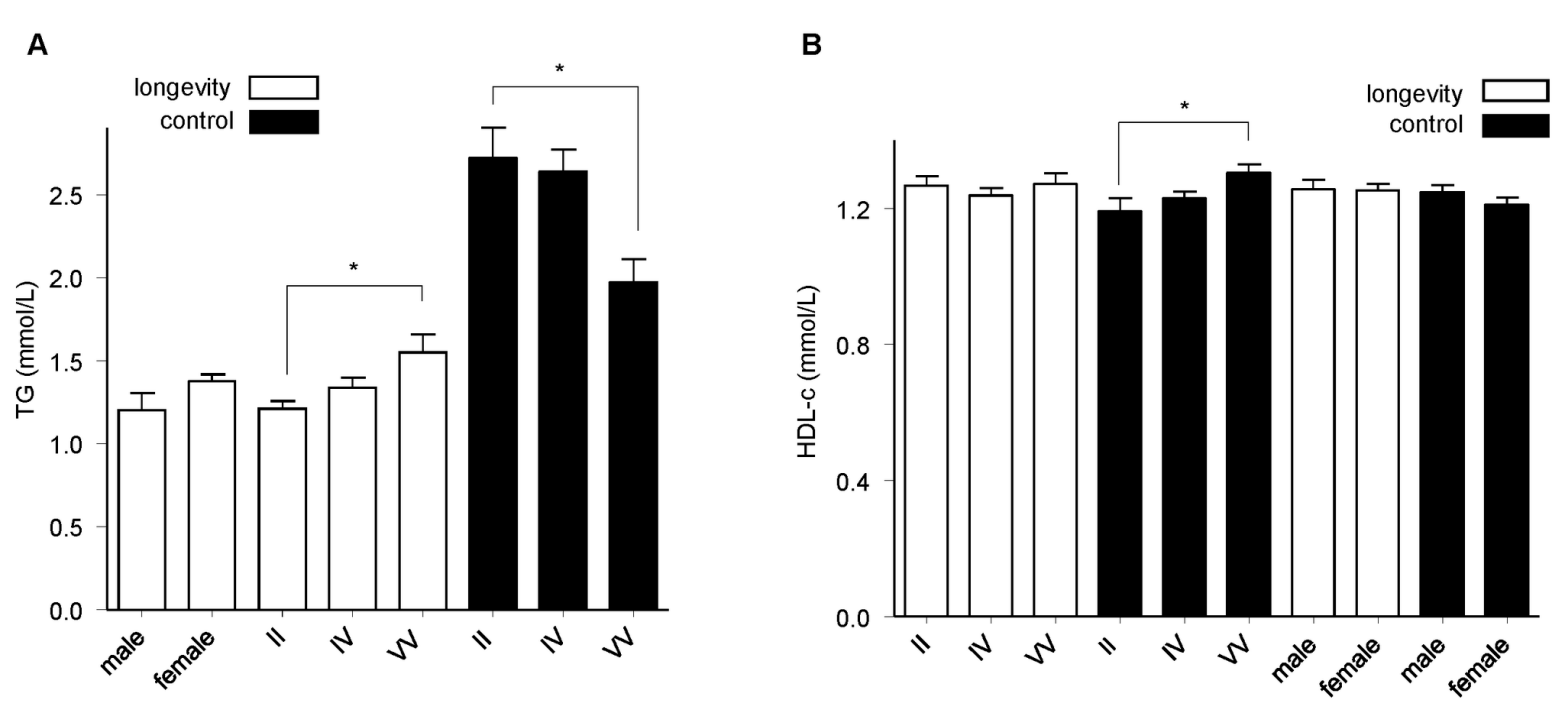
Comparison of serum TG and HDL-c among *CETP* I405V genotypes and genders in longevity and control subjects. (**A**) **TG (mmol/L); (B) HDL-c (mmol/L)**. *difference reached the significant level (*P*<0.05).

The demographic and metabolic characteristics of participants are shown in Table **1**. Fewer males were observed in the longevity group compared with the control group, which is in line with the worldwide gender imbalance in longevity. Power analyses showed that 86.0% power (additive) was required to detect a genotype relative risk of 0.5 at an alpha level of 0.05 for variants with a minor allele frequency (MAF) of 47.1% (HapMap Release 28), assuming a prevalence of longevity in Yongfu County of 0.0133%; 94.4% power (additive) was required to detect a genotype relative risk of 2.0. The *CETP* I405V distribution was in Hardy-Weinberg equilibrium in both groups (Table **2**). In the longevity group, One-way ANOVA test was conducted to compare the clinical parameters between genotypes and found that the triglyceride (TG) level was elevated in VV carriers compared to VI and II carriers (*P*_ANOVA_=0.016) (Figure **1A**). We did not identify any difference in HDL-c concentrations, among the *CETP* I405V genotypes in our longevity group (Figure **1B**). Bivariate Spearman correlation analysis indicated a decrease of total cholesterol (TC), TG, body mass index (BMI) and waist circumstances (WC) with increased ages in the longevity group (Figure S1).

**Table 1:** Demographic and metabolic traits between longevity and control groups.

Characteristics	Longevity (n=506)	Control (n=515)	
Gender (Male) (%)	26.5	67.0	^a^
Age (years)	92.45±3.57	41.40±10.54	^a^
BMI (kg/m^2^)	18.59±3.91	24.01±4.46	^a^
WC (cm)	76.75±9.39	82.67±9.65	^a^
Systolic BP (mmHg)	151.70±25.53	117.83±16.42	^a^
Diastolic BP (mmHg)	80.38±13.78	75.21±11.96	^a^
FPG (mmol/L)	5.68±1.46	6.17±2.17	^a^
TG (mmol/L)	1.33±0.89	2.52±2.04	^a^
TC (mmol/L)	4.75±1.07	4.98±1.27	^a^
HDL-c (mmol/L)	1.25±0.35	1.23±0.36	NS
LDL-c (mmol/L)	2.92±0.94	2.60±1.14	^a^

BMI-body mass index; WC- waist circumference; BP-blood pressure; FPG-fasting plasma glucose; TG- triglyceride; TC-total cholesterol; HDL-_C_- high density lipoprotein cholesterol; LDL-_C_- low density lipoprotein cholesterol

^a^ *P*<0.05 between groups, NS=not significant

**Table 2:** Genotype distribution of *CETP* I405V in longevity and control groups.

Group	HWE (P)	Genotype (%)			Allele (%)	
		VV	VI	II	V	I
Longevity (n=506)	0.861	84 (16.6)	242 (47.8)	180 (35.6)	410 (40.5)	602 (59.5)
Control (n=515)	0.247	110 (21.4)	270 (52.4)	135 (26.2)	490 (47.6)	540 (52.4)

Based on the additive (VV/VI/II), dominant (VV+VI/II) and recessive (VV/VI+II) models of inheritance, we conducted logistic regression analysis to evaluate the contribution of *CETP* 405V to longevity (Table **3**). Because of the gender imbalance in longevity, we performed a gender-stratification test and multiple-logistic regression under Model 1 (adjusted for gender) and Model 2 (adjusted for gender, apolipoprotein E (*APOE*) ε4, BMI and HDL-c). Consistently, 405V was suggested to be a risk allele (in Model 2: *P*_*additive*_=0.008; *P*_dominant_=0.008, OR_dominant_=0.673; *P*_*recessive*_=0.017, OR_recessive_=0.654). Moreover, the accumulated number of V allele was suggested to increase the risk against longevity as well (in Model 2: P=0.002, OR=0.734). Additionally, the interaction between 405V and *APOE* ε4 was considered a longevity risk (P=0.001, OR=0.530). Thus, not only was *CETP* 405V shown to be an independent longevity risk allele but synergy was also detected between *CETP* 405V and *APOE* ε4.

**Table 3:** Logistic regression analysis for the contribution of *CETP* 405V to longevity under different genetic models.

Variable	Stratification		Wald	*P*	OR (95%CI)
Gender		Male / Female	157.870	<0.001	0.177 (0.136-0.232)
*CETP*	overall	Additive(VV/VI/II)	11.293	0.004	NA
		Dominant(VV +VI/II)	10.419	0.001	0.643 (0.492-0.841)
		Recessive (VV/VI+II)	3.739	0.053	0.733 (0.535-1.004)
		Number of V allele	10.425	0.001	0.746 (0.624-0.891)
	Male	Additive(VV/VI/II)	16.440	<0.001	NA
	Female	Additive(VV/VI/II)	9.103	0.011	NA
Model 1^a^	overall	Additive(VV/VI/II)	11.146	0.004	NA
		Dominant(VV +VI/II)	8.321	0.004	0.650 (0.485-0.871)^a^
		Recessive (VV/VI+II)	6.439	0.011	0.640 (0.453-0.903)^a^
		Number of V allele	11.133	0.001	0.718 (0.591-0.872)^a^
Model 2^b^	overall	Additive(VV/VI/II)	9.560	0.008	NA
		Dominant(VV +VI/II)	6.996	0.008	0.673 (0.501-0.902)
		Recessive (VV/VI+II)	5.691	0.017	0.654 (0.462-0.927)
		Number of V allele	9.558	0.002	0.734 (0.604-0.893)
*APOE* interaction	overall	405V by ε4	10.851	0.001	0.530 (0.363-0.773)

Logistic regression analysis: risk (OR) of longevity, NA = not available

Dependent variable: longevity group (1) vs. control group (0); Independent variables: Gender (male: 1, female: 0)

^a^ Logistic model 1: adjusted for gender; ^b^ Logistic model 2: adjusted for gender, *APOE* ε4 number, body mass index and concentration of HDL-c

### Meta-analysis

#### Literature search and characteristics of eligible studies

**Figure 2:**
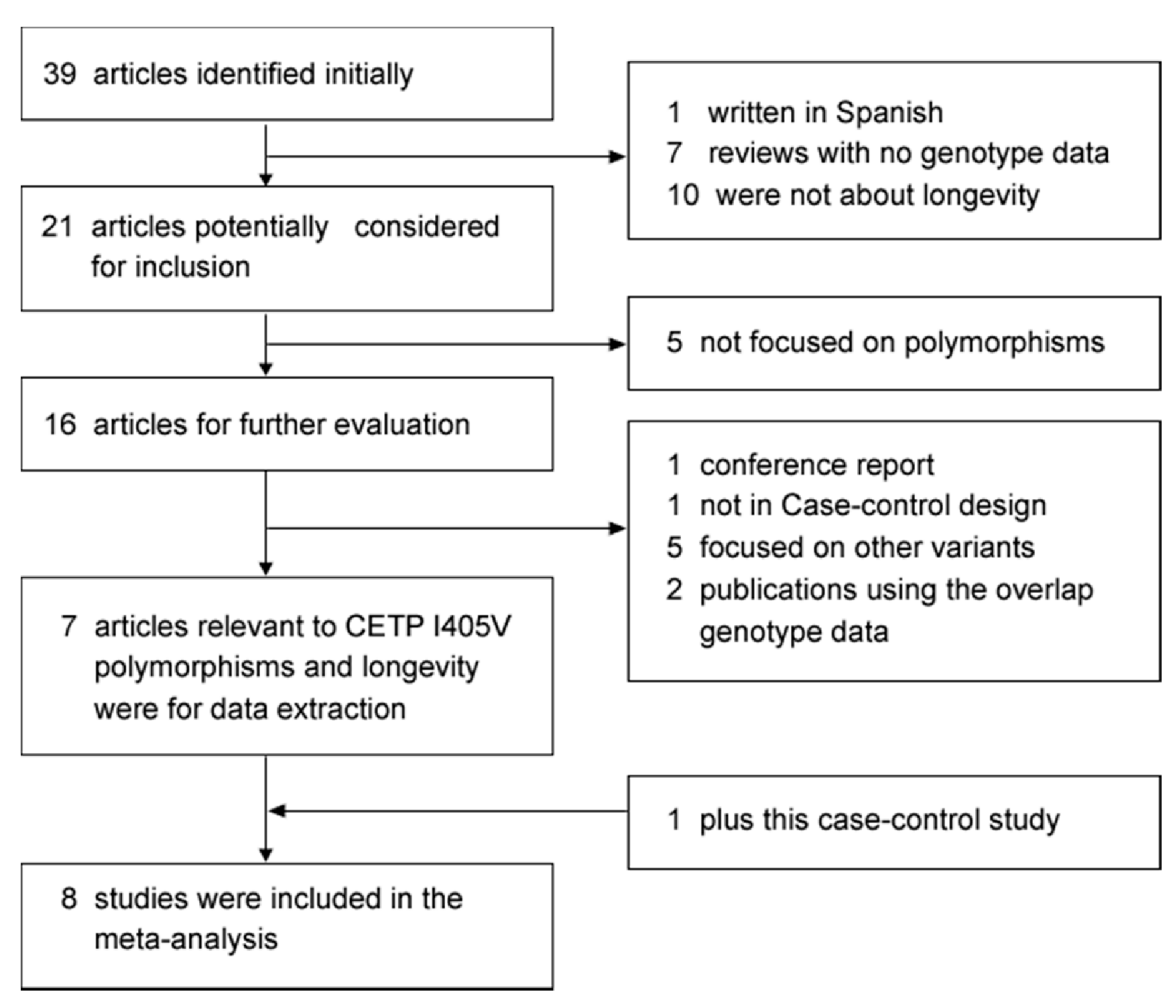
Flow diagram of study identification.

Figure **2** is a flow diagram of the strategy used to identify and search for inclusion in the meta-analysis. A total of 39 potentially relevant literature studies were retrieved by searching the electronic databases. Of these, 18 were excluded during a review of the abstracts, so the full text of 21 articles was evaluated. A further 14 studies were excluded because of missing genotype information, an absence of case-control design, publication data overlap, or inclusion of other *CETP* polymorphisms (Figure **2**). Accordingly, seven published studies were included in the current meta-analysis in addition to our case-control study described above [16,19–24]. This gave a total of 2,321 longevity subjects and 2,080 controls. The study characteristics are summarized in Table **4**. Among the eight case-control studies, there were one of Ashkenazi Jews, three of Europeans, three of East Asians and one of White Americans, consisting of two longevity subgroups. The genotyping methods used in the previous case-control studies were mainly based on restriction fragment length polymorphism analysis of polymerase chain reaction products (PCR-RFLP). The genotype distribution in the subjects of all studies was consistent with HWE.

**Table 4:** Characteristics of studies included in the meta-analysis.

Study	Ethnicity	Control					Longevity					*P* ^b^
		Age^a^	VV	VI	II	MAF	Age^a^	VV	VI	II	MAF	
Barzilai (2003) [16]	Ashkenazi Jews	71.3	11	75	43	0.38	98.2	37	72	47	0.47	0.003
Kolovou (2013) [19]	Europeans	60	9	45	43	0.32	93	23	97	75	0.37	0.584
Cellini (2005) [21]	Europeans	69.2	19	95	75	0.35	99.6	15	77	83	0.31	0.329
Vergani (2006) [22]	Europeans	31	7	61	32	0.38	89	17	44	39	0.38	0.022
Novelli-1^c^ (2008) [20]	White Americans	27.4	40	165	143	0.35	101.7	33	146	182	0.29	0.043
Novelli-2^c^ (2008) [20]	White Americans	27.4	40	165	143	0.35	95.9	41	149	171	0.32	0.214
Sun (2007) [24]	East Asians	67	6	31	16	0.41	97	23	92	76	0.36	0.385
Zhang (2011) [23]	East Asians	62.8	107	146	48	0.60	100.5	45	152	79	0.44	<0.001
This study	East Asians	41.4	110	270	135	0.48	92.5	84	242	180	0.41	0.003

^a^ mean age (years); ^b^ *P* value within each cohort estimated based on additive model (df=2); ^c^ The study was divided into two comparisons because it was consisted of 2 subgroup of longevity MAF: minor allele frequency; genotype (II, VI and VV) number, mean of age and MAF are taken from corresponding studies

#### Quantitative Synthesis

**Figure 3:**
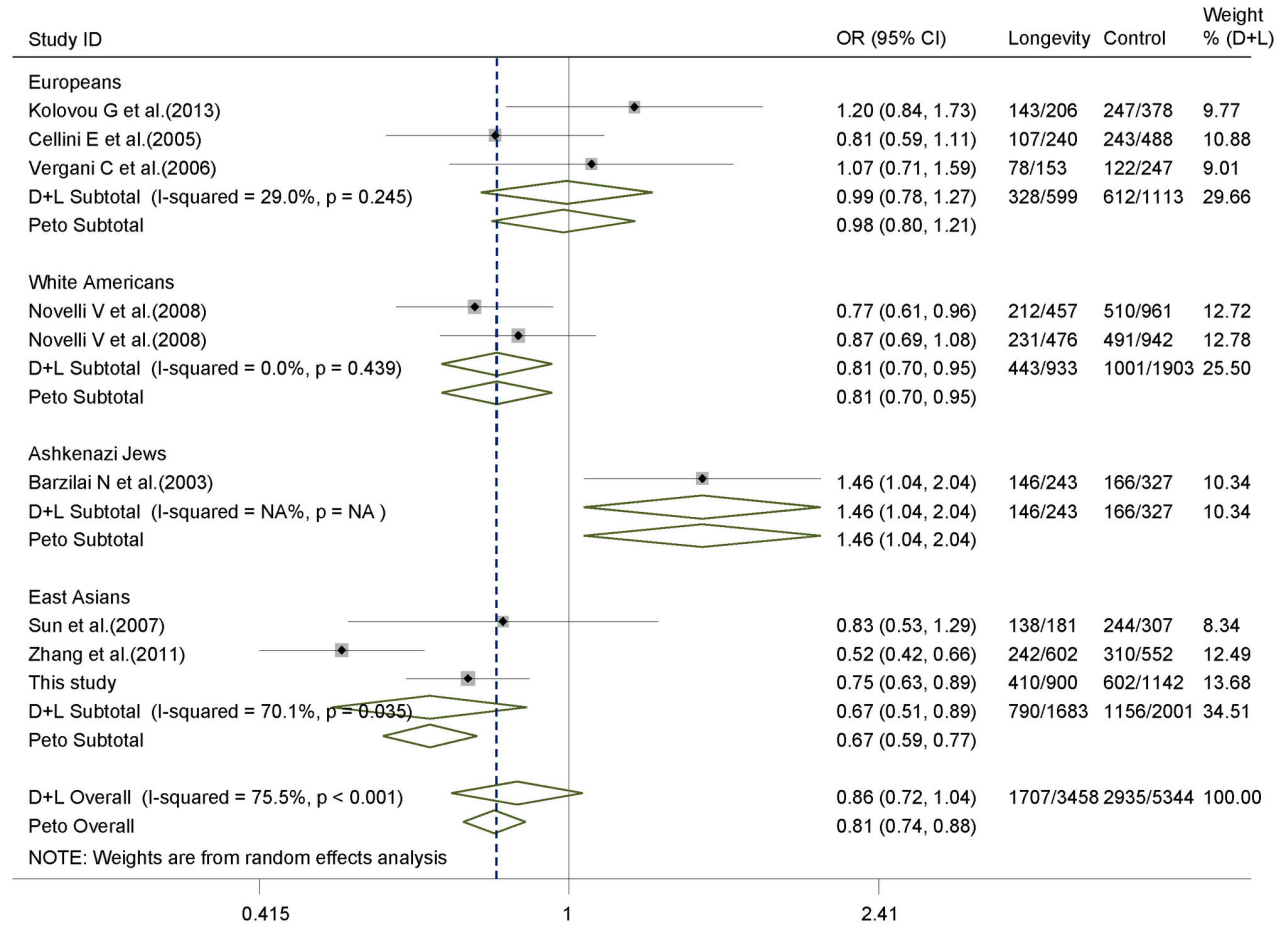
Forest plot (random effects model) describing the association of *CETP* I405V polymorphism with risk of longevity by Ethnicity in allele contrasts model. The *CETP* 405V was associated with decreased risk of longevity in allele contrasts model using random effects model by two methods, D+L and Peto, respectively. Each study is shown by the point estimate of the OR (the size of the square is proportional to the weight of each study) and 95%CI for the OR (extending lines). NA = not available.

The main results of the current meta-analysis and heterogeneity test are shown in Table 5. A total of nine datasets from eight studies were included in the analysis. Considering the power for the heterogeneity estimation, we calculated the Q and I-squared statistic in all subjects and found a significantly high heterogeneity in all genetic models (allele contrasts: *P*<0.001, I-squared=75.5%; dominant model: *P*=0.086, I-squared=42.3%; recessive model: *P*<0.001, I-squared=80.5%). The pooled OR (95%CI) was assessed according to the random-effects model (evaluated by D+L and Peto methods). Based on the Peto calculation for *CETP* 405V, we observed a significantly increased risk against longevity in all genetic models as shown in Figure **3**, Figure **S2** and Figure **S3** (allele contrasts: OR=0.81, 95%CI=0.74-0.88; dominant model: OR=0.72, 95%CI=0.64-0.82; recessive model: OR=0.80, 95%CI=0.67-0.96).

**Table 5:** Pooled and stratification analysis of the risk V allele of *CETP* I405V polymorphism on longevity.

Genetic model		N^a^	longevity	control	Q test		D+L	Peto	Begg’s Test	Egger’s Test
					*P*	I^2^ (%)	Pooled OR (95%CI)	Pooled OR (95%CI)	*P* ^e^	*P* ^f^
Allele contrasts^b^	overall	9	2321	2080	<0.001	75.5	0.86 (0.72-1.04)	0.81 (0.74-0.88) ^g^	0.251	0.138
	East Asians	3	973	869	NA	NA	0.67 (0.51-0.89) ^g^	0.67 (0.59-0.77) ^g^		
	Europeans	3	470	386	NA	NA	0.99 (0.78-1.27)	0.98 (0.80-1.21)		
	White Americans	2	722	696	NA	NA	0.82 (0.70-0.95)	0.82 (0.70-0.95)		
	Ashkenazi Jews	1	156	129	NA	NA	1.46 (1.04-2.04) ^g^	1.46 (1.04-2.04) ^g^		
Dominant^c^	overall	9	2321	2080	0.086	42.3	0.74 (0.62-0.88) ^g^	0.72 (0.64-0.82) ^g^	0.348	0.379
	East Asians	3	973	869	NA	NA	0.59 (0.48-0.73) ^g^	0.59 (0.48-0.73) ^g^		
	Europeans	3	470	386	NA	NA	0.88 (0.61-1.27)	0.87 (0.66-1.15)		
	White Americans	2	722	696	NA	NA	0.73 (0.59-0.90) ^g^	0.73 (0.59-0.90) ^g^		
	Ashkenazi Jews	1	156	129	NA	NA	1.16 (0.70-1.91)	1.16 (0.70-1.91)		
Recessive^d^	overall	9	2321	2080	<0.001	80.5	1.01 (0.66-1.55)	0.80 (0.67-0.96) ^g^	0.076	0.063
	East Asians	3	973	869	NA	NA	0.60 (0.33-1.11)	0.58 (0.45-0.73) ^g^		
	Europeans	3	470	386	NA	NA	1.36 (0.71-2.63)	1.30 (0.82-2.07)		
	White Americans	2	722	696	NA	NA	0.88 (0.63-1.23)	0.88 (0.63-1.23)		
	Ashkenazi Jews	1	156	129	NA	NA	3.34 (1.62-6.85) ^g^	3.34 (1.62-6.85) ^g^		

^a^Number of studies; ^b^ Allele V vs. I; ^c^ Genotype VV+VI vs. II; ^d^ Genotype VV vs. VI+II; ^e^ *P* value = Begg’s Test Pr>|z|; ^f^ *P* value = Eegg’s Test Pr>|t|; ^g^ *P* value reached the significance NA=not available

In the subgroup analysis stratified by ethnicity, a significantly protective contribution for longevity was only found in Ashkenazi Jews (allele contrasts: OR=1.46, 95%CI=1.04-2.04; recessive model: OR=3.34, 95%CI=1.62-6.85), not in other ethnicities. The Peto method found a significantly increased risk against longevity for *CETP* 405V in East Asians in all genetic models. This was also indicated by the D+L method in allele contrasts and the dominant model. No significant stable contribution was found with respect to Europeans or White Americans.

One-way sensitivity analysis was conducted to evaluate the stability of the meta-analysis and the statistical significance of the results was not altered when any single study was omitted (data not shown).

#### Publication Bias Analysis

**Figure 4:**
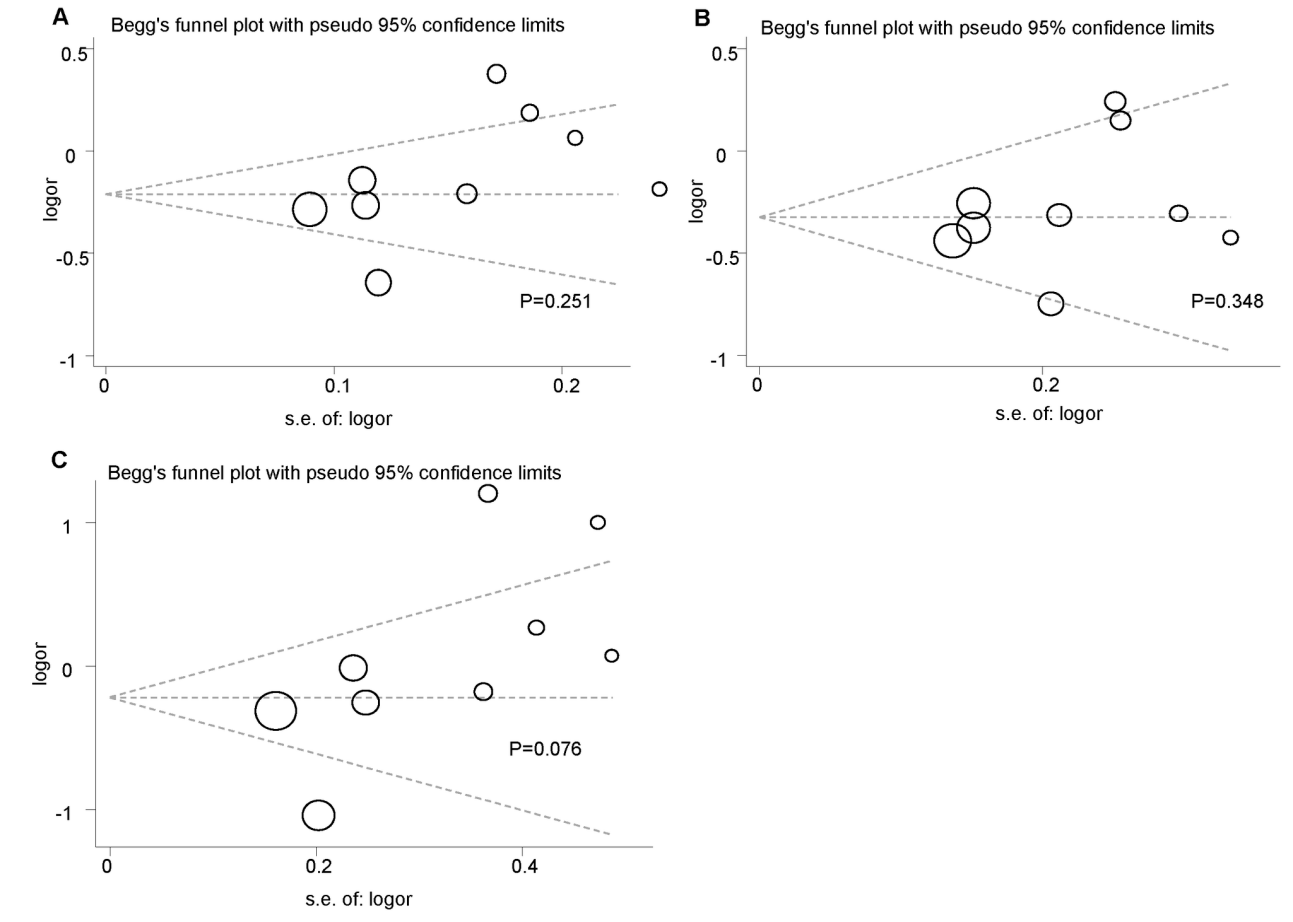
Begg’s funnel plot of *CETP* I405V polymorphism to detect publication bias. (**A**) **allele contrasts **(**405V vs**. **405I**)**; **(**B**) **dominant model **(**405VV+VI vs**. **II**)**; **(**C**) **recessive model (405VV vs**. **VI+ II).** Each circle represents an individual study for the indicated association and whose size proportionally represents the weight of each study. logor, natural logarithm of OR; s.e., standard error; Perpendicular line, mean effect size.

Begg’s funnel plot, Begg’s test and Egger’s test were performed to assess the publication bias of the literature. As shown in Figure 4, the shapes of the funnel plots were symmetrical for *CETP* I405V in allele contrasts, dominant and recessive genetic models and this was statistically supported by the Begg’s and Egger’s test (allele contrasts model: *P*_*Begg’s*_=0.251, *P*_*Egger’s*_=0.138; dominant model: *P*_*Begg’s*_=0.348, *P*_*Egger’s*_=0.379; recessive model: *P*_*Begg’s*_=0.067, *P*_*Egger’s*_=0.063) (Table **5**).

## Discussion

Nowadays, to understand the genetics and consequently the biological processes involved in aging and longevity is one of the greatest challenges. Although the environmental factors have led to an increase in life span, there is ample evidence that genetic factors are involved in extreme longevity and other organisms [34]. Additionally, the global sample size of longevity is relatively limited compared with those complex diseases, which restricts the power for the genome-wide replication study to a great extent. Realistically, most of the population genetic works have taken place in candidate genes rather than by genome-wide analysis. Accordingly, considering the important roles of CETP in lipid metabolism and longevity, we conducted an association analysis between a functional polymorphism *CETP* I405V and longevity in Chinese as the largest research in East Asians by far, followed by a meta-analysis.

*CETP* I405V is a common SNP (rs5882) arising from A-to-G substitution in exon 14, corresponding to a change from isoleucine to valine at codon 405. 405V is the minor allele occurs at a frequency of >30% in most populations (Table **4**). In an attempt to arrive at a more definitive conclusion about the associations between I405V polymorphisms and longevity in Chinese, we performed a case-control study and a subsequent meta-analysis.

The present study initially compared the clinical parameters between longevity and control groups, which might potentially confound the genetic contributions. Spearman correlation analyses indicated a decrease in TC, TG, BMI and WC with increased age in the longevity group, not the control group, which could be explained by the natural selection of death related diseases (Figure **S1**). Because of the known importance of *CETP* I405V in the regulation of HDL-c, we compared HDL-c level among different I405V genotypes. HDL-c was significantly elevated with increasing numbers of 405V allele in the control group (*P*<0.05), although this was not replicated in the longevity group (Figure **1B**). Moreover, the TG level was also increased in VV carriers compared to VI and II carriers, which was dependent on age group, while, conversely, V allele carriers had lower TG levels in the control group. Perhaps because of the population-specific distribution of HDL-c and TG in our longevity group, our case-control study indicated a significant risky contribution of *CETP* 405V against longevity in each of the genetic models adjusting for gender, *APOE* ε4, BMI and HDL-c confounding; this contradicts the protective results previously reported in Ashkenazi Jews [16] (Table **3**). Our data also indicated a synergy between *APOE* ε4 and 405V against longevity.

Certain factors could have influenced the findings of our case-control study. First, there might have been a complex mechanism between clinical parameters and longevity in that subjects who met the criteria for longevity demonstrated a diverse clinical status. Second, the above-mentioned diversity of clinical parameters could have been obtained by centenarians suffering a difficult early life in terms of environmental, lifestyle, and social changes that could not be matched in modern-day populations [4]. Third, the MAF differences of *CETP* I405V and its contributions to longevity might be partly attributed to ethnicity. Besides specific life-style and social changes, the genetic background between ethnicities proved to be more different than we had first thought.

Meta-analysis is considered to be a powerful tool for pooling the results from different studies in complex traits, because of the relative improved statistical power achieved with larger sample sizes [35]. We therefore conducted a meta-analysis of seven published articles from different populations and the results from our case-control study by a random-effects model. Based on all three genetic models using the Peto method, *CETP* 405V was indicated as a longevity risk allele in the overall populations (Table 5). When stratified by ethnicity, only the study of Ashkenazi Jews indicated a protective role for 405V in longevity, which was not confirmed in other ethnicities. Notably, three Chinese studies indicated a higher MAF of *CETP* 405V in the control group than other ethnicities, and this was also supported by corresponding data in the International HapMap project (Release28). Generally, both D+L and Peto methods suggested a significantly increased longevity risk for *CETP* 405V in East Asians for all genetic models, but not in Europeans or White Americans. The inconsistencies in the results from the studies cited might be partially explained by differences in study design, sample size, genetic background, age, gender and synergetic effects with other genetic determinants. By analyzing linkage disequilibrium patterns using international HapMap projects data, we observed obvious differences between ethnics. Besides, although *CETP* I405V is a missense mutation, the modeling of the three-dimensional structure of CETP indirectly based on that of the evolutionarily-related bactericidal/permeability inducing protein indicated that the I405V polymorphism was too distant from the lipid binding pocket to affect lipid binding directly [15]. Thus, while I405V represents a conservative amino acid change, it might be in linkage disequilibrium with another closed gene that directly influences the HDL-c, TG and longevity risk. If this were the case, the intensity of linkage disequilibrium would vary according to different genetic backgrounds; this could be verified by next-generation sequencing techniques.

Many factors could have interfered with our meta-analysis. First, the number of recruited studies and sample size in each study is relatively limited, which might make it easier for distribution skewness and reduce the population representativeness. Second, the inter-study heterogeneity is potentially a significant problem which could be due to the differences in sample selection, genotyping quality, and healthy status confounding which is related to longevity, which could not be matched well among studies without a systematic pre-design, such as lipid levels and other risk factors. Although the ethnic-specific risky association reported here didn’t reach an extreme significance yet, the findings are important to report so that more attention could be paid to the ethnicity difference when further applying *CETP* 405V to the healthy status and longevity prediction. Besides, the data from HapMap (Release 18) indicated a diversity of the baseline of *CETP* I405V among ethnicities. In Chinese and Japanese, subjects have higher frequencies of *CETP* 405V than the Europeans and Tuscans in Italy. And subjects from Kenya and African ancestry Americans have the highest frequencies. The population migration might be involved which could be further invested by molecular anthropology. Besides, the known differences in environment, diet pattern, lifestyle and body composition between Asian and other ethnicities should be considered as well. Additionally, no difference of HDL-c was observed between longevity and control group in our subjects, indicating a potential diverse of contribution of *CETP* 405V to longevity.

In conclusion, our results suggests *CETP* 405V as a risky allele against longevity in Chinese. Since the data of *CETP* I405V with longevity in East Asians is limited, further replication studies in Japanese and Koreans should be conducted; particularly focusing on the effects of gene-gene and gene-environmental interactions. More importantly, further long-term prospective studies with larger samples between offspring of centenarians and their age-matched spouse or neighbor are necessary to support the transfer of genetics from their prolong-lived parents, especially concerning the impact on lipoprotein subclasses and CETP concentration.

## Supporting Information

Checklist S1 **PRISMA checklist.**
(DOC)

Figure S1 **Decrease in TC, TG, BMI and WC with the increasing of ages in longevity group.**
(DOC)

Figure S2 **Forest plot (random effects model) describing the association of *CETP* I405V polymorphism with risk of longevity by Ethnicity in dominant model.**
(DOC)

Figure S3 **Forest plot (random effects model) describing the association of *CETP* I405V polymorphism with risk of longevity by Ethnicity in recessive model.**
(DOC)
