# Supplementary material for: Trans-Ethnical Shift of the Risk Genotype in the CETP I405V with Longevity: A Chinese Case-Control Study and Meta-Analysis
Source: PLoS One. 2013 Aug 15;8(8):e72537. doi: 10.1371/journal.pone.0072537 (PMC3744487; doi:10.1371/journal.pone.0072537)
Supplement: Figure S1 — Decrease in TC, TG, BMI and WC with the increasing of ages in longevity group. (DOC) [file pone.0072537.s002.doc]

**Supplementary**

**
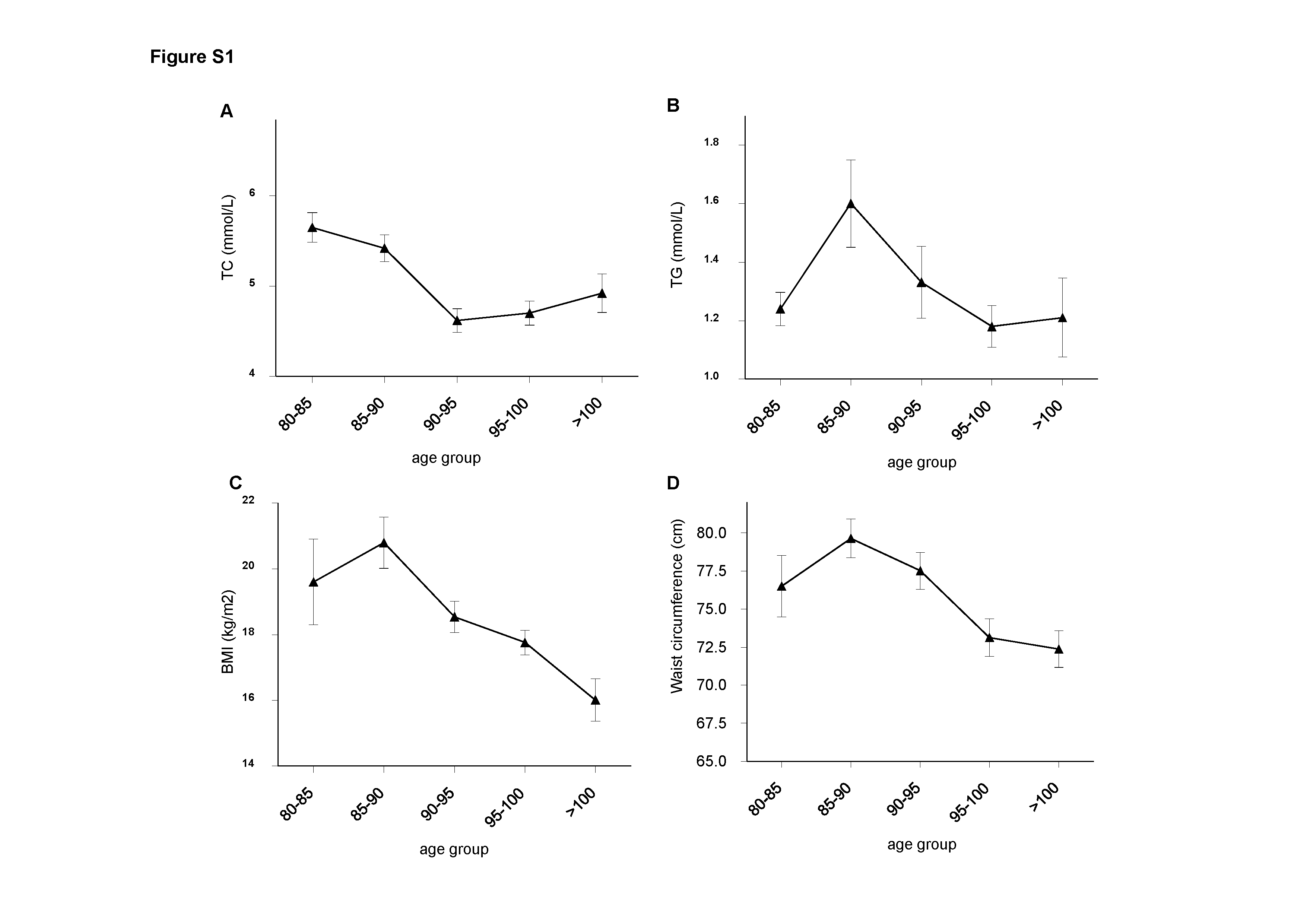
**

**Figure S1.** **Decrease in TC, TG, BMI and WC with the increasing of ages in longevity group.**
