# Supplementary material for: Trans-Ethnical Shift of the Risk Genotype in the CETP I405V with Longevity: A Chinese Case-Control Study and Meta-Analysis
Source: PLoS One. 2013 Aug 15;8(8):e72537. doi: 10.1371/journal.pone.0072537 (PMC3744487; doi:10.1371/journal.pone.0072537)
Supplement: Figure S2 — Forest plot (random effects model) describing the association of CETP I405V polymorphism with risk of longevity by Ethnicity in dominant model. (DOC) [file pone.0072537.s003.doc]

**Supplementary**

**
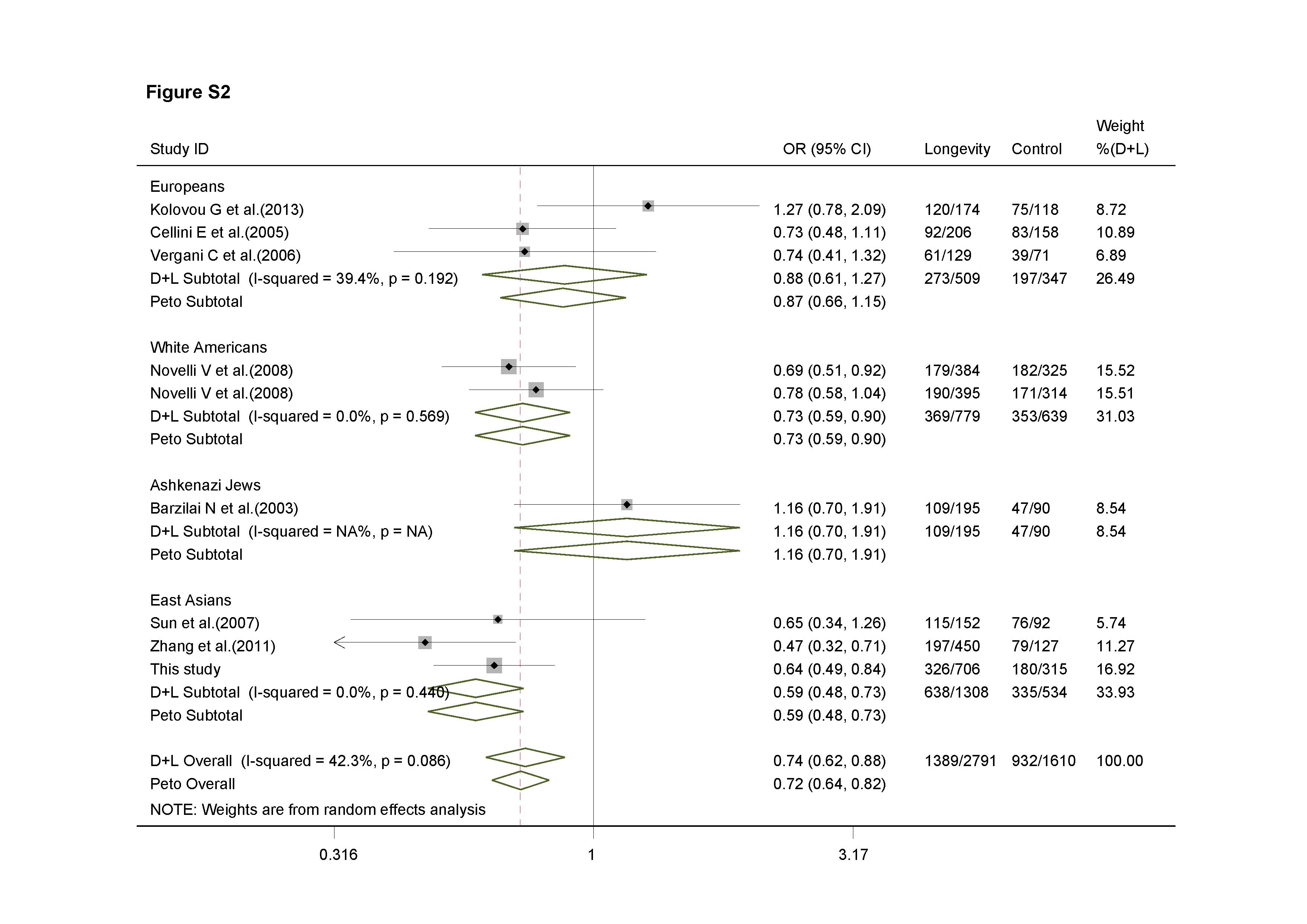
**

**Figure S2. Forest plot (random effects model) describing the association of CETP I405V polymorphism with risk of longevity by Ethnicity in dominant model.**

The CETP 405V was associated with decreased risk of longevity in dominant model using random effects model by two methods, D+L and Peto, respectively. Each study is shown by the point estimate of the OR (the size of the square is proportional to the weight of each study) and 95%CI for the OR (extending lines).
